# Electrochemically synthesized highly crystalline nitrogen doped graphene nanosheets with exceptional biocompatibility

**DOI:** 10.1038/s41598-017-00616-8

**Published:** 2017-04-03

**Authors:** Deepa Suhag, Arun Kumar Sharma, Satyendra K. Rajput, Gajender Saini, Sandip Chakrabarti, Monalisa Mukherjee

**Affiliations:** 1 Biomimetic and Nanostructured Materials Research laboratory, Amity Institute of Biotechnology, Amity University Uttar Pradesh, Sector-125, Noida, 201303 India; 2 Amity Institute of Pharmacy, Amity University Uttar Pradesh, Sector-125, Noida, 201303 India; 3 Advance Instrumentation Research Facility, Jawaharlal Nehru University, New Delhi, India; 4 Amity Institute of Nanotechnology, Amity University Uttar Pradesh, Sector-125, Noida, 201303 India

## Abstract

This work reports first electrochemical preparation of exceptionally biocompatible, highly crystalline, and well exfoliated nitrogen doped graphene nanosheets (eNGS) from carbon nanosheets for the development of mighty platforms in the field of modern biosensing and other biological applications for human welfare. eNGS displayed exceptional biocompatibility. Administration of the as-synthesized eNGS to rat models did not lead to any significant deviation or inimical consequences in its functional observation battery (FOB) tests, GSH levels or the histology of the vital organs of the rat models. The pictomicrographs of myocytes nuclei and myofibrillar for heart, hippocampus (CA1) section for brain, central vein, and hepatocytes for liver and parenchyma, tubules and glomeruli for kidney also remained unaffected. Moreover, the resultant nanoelectrocatalyst displayed enhanced electrochemical performance towards real-time sensing of dopamine (DA) from human urine sample in the presence of interferences, such as ascorbic acid (AA) and uric acid (UA).

## Introduction

Single- and multilayer carbon materials have drawn much recent attention owing to their unique free standing two dimensional (2D) structures. Graphene is one of the most commonly exploited carbon materials in the fields of electronic devices^1, 2^, catalysts^3–5^, sensors^6, 7^, energy conversion^8^, and storage devices^8^. However, the potential applicability of graphene is sometimes restricted due to its high cost of production and low yield, because of which graphene oxide (GO) is used as a starting material for graphene based applications. Several methods have been employed to synthesize GO sheets such as chemical vapour deposition (CVD)^9^, epitaxial growth by ultrahigh vacuum graphitization^10^, chemical oxidation of graphite and further reduction^11^. In recent years, attempts have been made to produce graphene flakes using electrochemical techniques. Electrochemical route is an effective method to permute electronic states by tailoring the potential in order to alter the Fermi energy levels of the modified electrode surface^12^. The biggest advantages of electrochemical techniques over other methods are control over the synthesis procedure (e.g., allows thickness control by adjusting the potential), mild reaction conditions along with being economic, facile, green, and non-destructive^13^. Our group prepared highly biocompatible, nitrogen doped graphene nanosheets (eNGS) *via*. electrochemical reduction of carbon nanosheets (CNS). CNS are a new class of carbon materials which comprise of few layered graphene films. The as-synthesized CNS possesses exceptional thermal conductivity and electrical conductivity along with high surface to volume ratio, thin edges, lightness, and a large number of edge plane defects. CNS are also known to embody high electrochemical stability as they do not gather together and are grown *via*. catalyst free procedures^14–16^. Until now CNS has been synthesized by either top-down or bottom up method and has also been subjected to calcination and pyrolysis, resulting in decomposition of oxygen containing functionalities and collapse of CNS structure thereby leading to a marked decrease in the in-plane sp^2^ domain size. Therefore, in order to restore the graphitic structure of CNS as well as to enhance its conductivity and electrocatalytic activity, we considered synthesizing our graphene nanosheets *via*. electrochemical reduction of CNS. This one-step electrochemical synthesis of eNGS banishes the potential contaminants which occur in case of chemical reduction. Additionally, the successfully eliminated oxygen moieties along with other morphological modifications represents a facile, green, and efficient route to fabricate high performance electrocatalytic biosensors which would also display biocompatibility due to nitrogen incorporation into the basic framework of eNGS^17, 18^. Recently, our group reported the hydrothermal preparation of nitrogen doped graphene nanosheets (NGS), which were used as an electrode material to selectively determine dopamine (DA), and demonstrated exceptional potential separation which indicates the excellent selectivity of the system of DA and uric acid (UA)^19^. In spite of the superior electrocatalytic properties of NGS along with their high repeatability and stability as an electrochemical nanoelectrocatalyst, we were unable to determine DA in the presence of ascorbic acid (AA). Alternately, Chen *et al*. also demonstrated the use of 3D graphene foam as a novel electrode architecture for selective detection of DA with remarkable sensitivity and lower detection limit. Notably, their material as well could selectively detect DA only in the presence of UA^20^.

Adoption of DA as an analyte of interest is also driven by its pivotal role as a key neurotransmitter in the human central, nervous, renal, and cardiovascular systems^21, 22^. Our choice of detection and quantification of DA concentration are pertinent for diagnosis, monitoring, and prevention of various pathological disorders such as, Parkinson’s disease and Huntington’s disease^21, 22^. Therefore, it is very imperative to detect the precise concentration of DA in the biological systems. Moreover, AA and UA are the major and most prominent interfering species which hinder the selective detection of DA. This is ascribed to their co-existence with DA in the real biological samples and their similar oxidation peak potentials, which thereby lead to overlapping of the peaks. To overcome this predicament and establish selective and sensitive procedures for the detection of DA, numerous electroactive materials, acquiring catalytic effects on DA oxidation have been employed to attain the successfully modified electrode materials.

In the present study, we for the first time report a facile and single step electrochemical synthesis of eNGS. The newly prepared, highly crystalline, conductive and electrocatalytic eNGS was then employed to electrochemically detect DA with greater sensitivity and selectivity. Electrochemical sensors based on graphene have been reported as attractive biosensing platforms because of their good sensitivity, however with drawbacks such as poor selectivity, compromised biocompatibility, low long term stability, repeatability, and high charge transfer resistance. We therefore prepared eNGS, where majority of oxygen containing functionalities are irreversibly reduced resulting in restoration of its graphitic structure and an increase in its conductivity. Subsequently, eNGS was also employed for real-time detection of DA from human urine samples in the presence of interferences, such as AA and UA. This work also consists of a battery of biocompatibility assays for the as-synthesized eNGS, thus establishing their broad spectrum, practical applications in complex biological systems.

## Materials and Methods

### Synthesis and preparation of Pt-eNGS electrode

CNS was synthesized as reported in our previous work^19^. A Pt- electrode acquired from Gamry Instruments, USA, with geometrical area of 0.071 cm^2^ (3 mm in diameter) was used for sensor preparation. Prior to use, Pt- electrode was polished with 0.1 µm, 0.3 µm and 0.5 µm of alumina/DI water slurries on a polishing cloth to obtain a mirror like finish. The polished electrode was then rinsed thoroughly with ethanol solution, acetone solution, and DI water in ultrasonic bath. Subsequently, the electrode was then sonicated in acetone and DI water, respectively. The electrode was then air dried under ambient conditions. eNGS was synthesized by taking 1 mg/ml of CNS and drop coating 5 µl of the sonicated CNS solution onto a conductive electrode surface (e.g. Pt-) and dried under ambient conditions. All voltammetric measurements were carried out in a three electrode cell using Gamry Reference 600 Potentiostat/Galvanostat/ZRA where a Pt- wire was taken as a counter, an Ag/AgCl electrode as a reference and the CNS/eNGS modified Pt- electrode as a working electrode. An aqueous solution containing 0.1 M phosphate buffered saline (PBS) was used as an electrolyte solution. The I-V curves were measured using a Keithley 6517B Electrometer/High resistance meter.

The experimental protocol (CPCSEA/AIP/2015/03/005) used in the present study was approved by the Experimental protocol for the present study was approved by ‘Institutional Animal Ethics Committee’ Amity Institute of Pharmacy (Block-A), Amity University, Noida, Uttar Pradesh (Approval No: CPCSEA/AIP/2015/03/005) as per the guidelines of ‘Committee for the Purpose of Control and Supervision of Experiments on Animals’ under Chapter 4, Section 15(1) of the Prevention of Cruelty to Animals Act 1960’ (CPCSEA; Ministry of Environment, Forest & Climate Change, Government of India, (Animal Welfare Division), 5th Floor, ‘Vayu’ Wing, Indira Paryavaran Bhawan, Jor Bagh Road, New Delhi - 110 003)’. Wistar albino rats of either sex, weighing ~250 g were acclimatized in the ‘Institutional animal house’ and maintained on free access of food and water. They were exposed to normal day and night cycles. 6 rats (either sex) were divided into 2 groups; 1 = Normal control (No treatment shall be given/normal saline) and 2 = Treated with eNGS (300 mg/Kg/*p.o*). Functional parameters (FOB) were evaluated after 30 minutes of eNGS administration, whereas biochemical and histological assessments were carried at the end of 14th day.

### Functional observational battery

Functional observational battery (FOB) was analysed for any detrimental effects of eNGS on behavioural alterations. Animals including normal control and eNGS treated were examined for FOB activity 30 minutes after eNGS administration. Responses were scored on a 5-point scale (1 = lowest activity and 5 = highest activity). FOB was prepared to reveal early undesirable effects of eNGS on central nervous system, which includes various behaviour parameters^23^.

### Sample preparation and assay of reduced glutathione (GSH)

Rats were dissected at the end of the experiment and the tissues of vital organs (brain, liver, heart, and kidney) were collected for biological evaluations. The tissues were homogenized with an KINEMATICA AG - POLYTRON® homogenizer (Bohemia, NY 11716 USA) in ice-cold 0.1 M phosphate-buffered saline (PBS, pH 7.4) containing 0.1 mM EDTA. Homogenates were centrifuged at 10,000 × g for 15 min at 4 °C and the supernatants were collected and stored at −20 °C. Similar sample preparations were performed for all the tissues. The concentration of GSH was measured by using commercially available kit (Cayman Chemical Company, 1180 East Ellsworth Road, Ann Arbor, MI 48108 USA). The assay optimized enzymatic recycling method for the quantification of GSH^24^.

Briefly, tissues were homogenized with 10 times (w/v) 0.1 sodium phosphate buffer (pH 7.4). This homogenate is then centrifuged with 5% trichloroacetic acid to separate the proteins. 50 μl of the centrifuged sample was added to 150 μl of reaction mixture including 0.4 M 2-(N-morpholino) ethane-sulfonic acid, 0.1 M phosphate (pH 6.0), 2 mM EDTA, 0.24 mM NADPH, and 0.1 mM 5, 5′-dithiobis-2-nitrobenzoic acid (DTNB). The mixture was vortexed and the absorbance was read at 412 nm within 25 min. All samples were analysed in triplicates and GSH level was stated in μmol/g tissue. The GSH levels reveal the redox status of tissue which concludes its detoxification ability.

### Histological assessment

At the end of experimental protocol, all animals from each group were sacrificed. Immediately, dissected vital organs (brain, heart, liver and kidney) were stored in 10% neutral formalin solution and embedded in paraffin wax. The sections were stained with hematoxylin and eosin. The stained sections were observed under light microscopy.

### Biochemical Assays

For estimation of biochemical parameters, blood was collected and centrifuge at 5000 rpm for 15 minutes at 4 °C. Serum was collected and used for different biochemical evaluations. Estimation of serum TNF-α and IL-6, IL-10, TBARS and lipid profile (total cholesterol, TC; high density lipoprotein, HDL; low density lipoprotein, LDL; triglyceride, TG) were estimated by using commercially available ELISA kits as discussed in the manual. Kits for the estimation of TNF-α and IL-6, IL-10 were purchased from RayBiotech, Inc. 3607 Parkway Lane, Suite 200, Norcross GA 30092, whereas kits for TC, HDL, LDL, and TG were procured form ERBA Diagnostics, Inc. 2140 North Miami Avenue Miami, FL 33127 USA and TBARS was measured as described in previous study^25^.

## Results and Discussion

### Electrochemical Synthesis of eNGS

**Figure 1:**
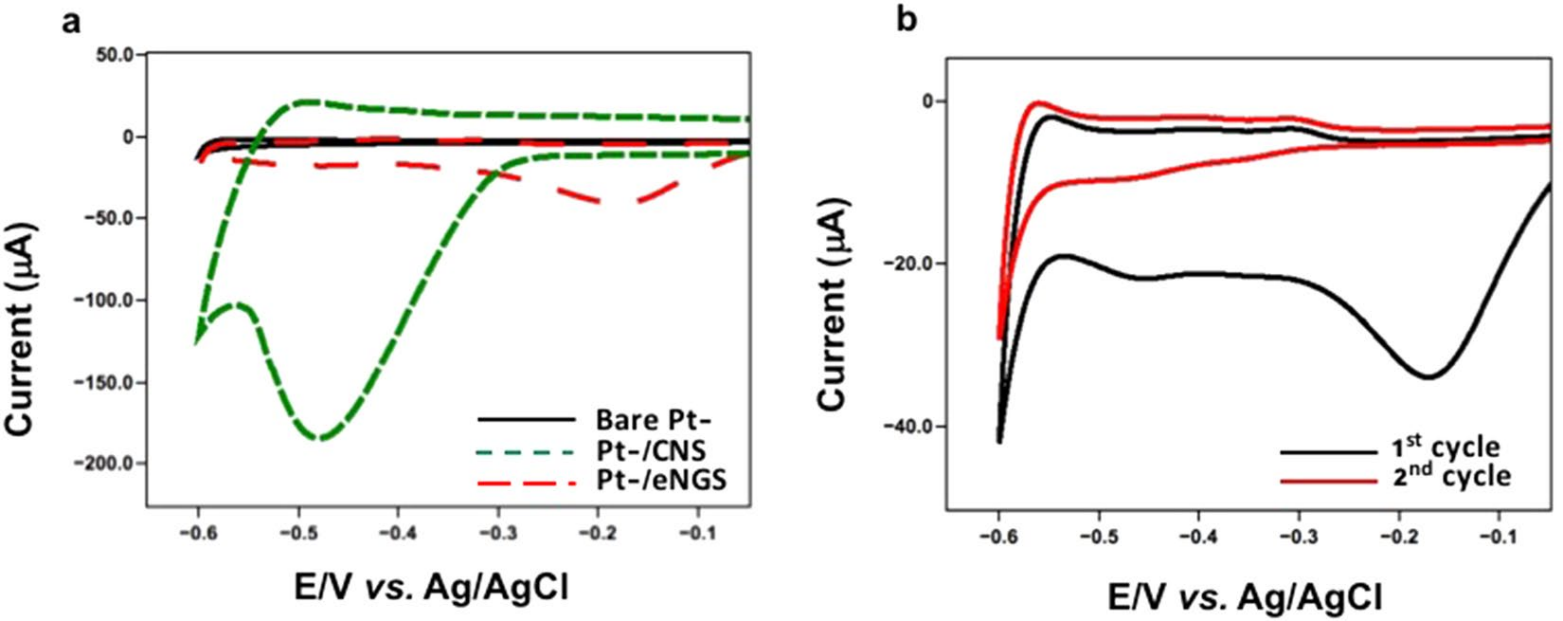
(**a**) Electrochemical synthesis of eNGS, and (**b**) Irreversible electrochemical reduction of eNGS in PBS (pH 4.5/saturated with N_2_ gas). Scan rate - 20 mV/s.

Figure 1a shows the cyclic voltammogram of the CNS-modified Pt-electrode in N_2_ saturated PBS (pH 4.5) solution. The comparative cyclic voltammogram of the CNS/eNGS modified Pt-electrode displayed a distinctive peak at −0.48 V for CNS, whereas in case of eNGS, a shift in peak towards a lesser negative potential from −0.48 V to −0.17 V was observed, thereby indicating the successful reduction of CNS to form eNGS. Similar results have been obtained by other groups for graphene and carbon nanotubes^26^. The electrochemical reduction of CNS to form eNGS was carried out by scanning in the potential window of 0 V to −0.6 V with respect to Ag/AgCl at a scan rate of 20 mV/sec. The peak observed at −0.17 V in the first scan cycle indicates the successful electrochemical reduction of CNS to form eNGS. The second scan at the same scan rate did not result in a peak at the same potential suggesting that the surface oxygenated species on the CNS surface have been completely reduced and that the electrochemical reduction of CNS to eNGS is an irreversible process^27, 28^. This large reduction current for eNGS was due to the reduction of surface oxygen groups (Fig. 1b)^12^.

**Figure 2:**
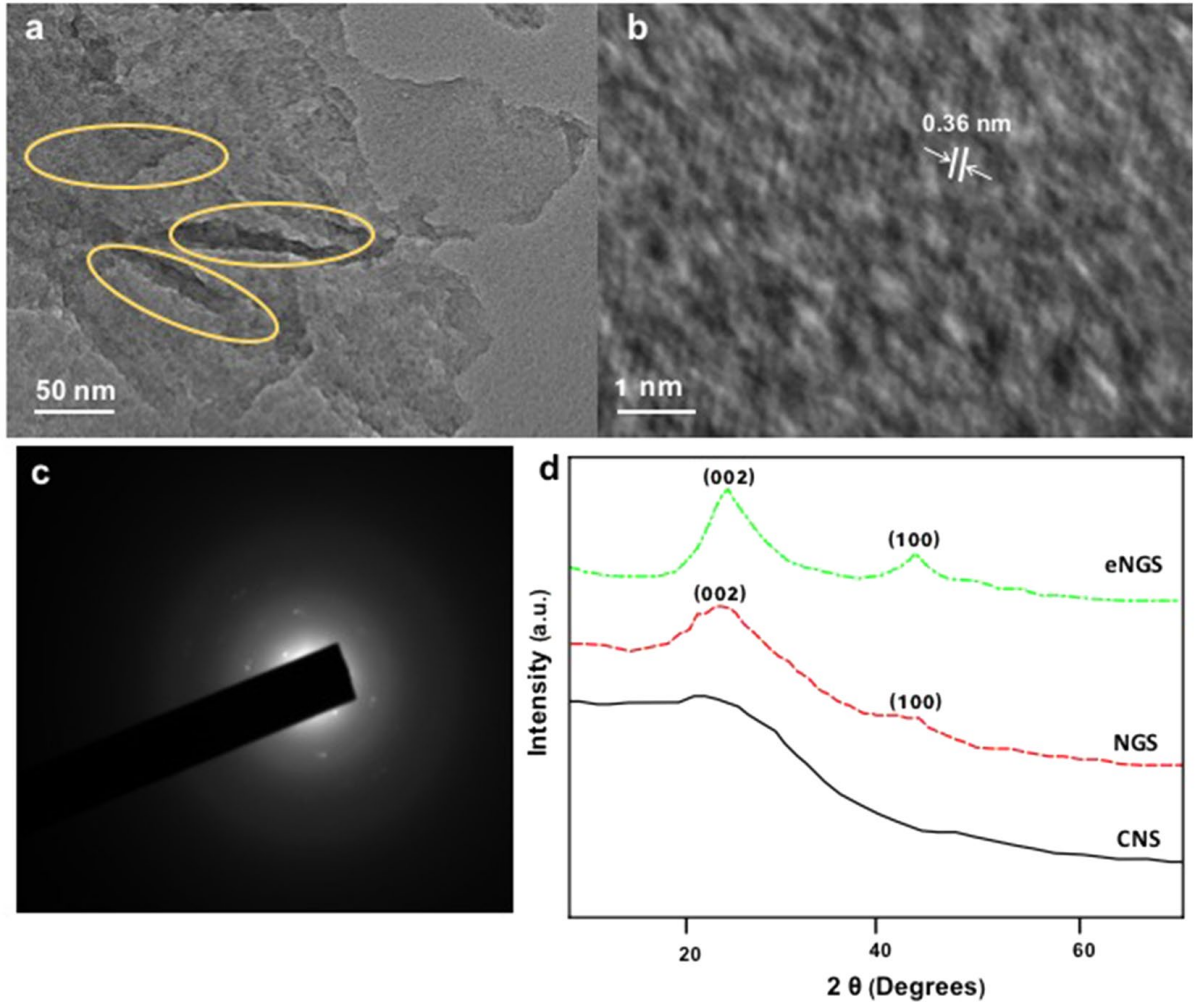
(**a**) Surface topographies of eNGS by HRTEM (yellow circles highlight the wrinkled and folded areas in the sheets) (**b**,**c**) SAED patterns of eNGS and (**d**) X-ray diffraction patterns of eNGS, NGS, and CNS.

High resolution TEM images of ultrasonically exfoliated eNGS exhibits typical wrinkled sheet like structures where sheets were seen to be exfoliated into very thin layers (Fig. 2a). The wrinkling observed in eNGS is caused by interlocking of carbon nanosheets, resulting in exceptional mechanical strength, increased surface-to-volume ratio, and a decrease in surface energy^29, 30^. The existence of distinct lattice lines on the enlarged HRTEM (Fig. 2b) displayed that wrinkled eNGS sheets are crystalline in nature. These electrochemically exfoliated eNGS were found to be ~1.2 nm thick sheets with lateral dimension of ~120 nm (Figure S2). The Selected Area Electron Diffraction (SAED; inset Fig. 2b) pattern of the as-synthesized sample comprises of diffraction points ascribed to graphene. The d-value measured from the spots is ~0.365 nm, representing a layered structure growth of graphene like nanosheets along (002).

The SAED pattern consist of more than one spot at every diffraction point which could be due to back-folding of edges and overlapping of graphene layers^31^. The diffraction points along with the rings are observed in the SAED pattern of eNGS. In SAED, the clearly-defined diffraction spots and rings are completely indexed to typically hexagonal lattice of carbon in NGS, thereby confirming the presence of crystallinity in eNGS synthesized *via*. electrochemical reduction of CNS.

Furthermore, XRD measurements were employed to evaluate the degree of exfoliation of the eNGS. The XRD patterns of CNS displayed no diffraction peaks, thereby highlighting the amorphous nature of the precursor material. However, our previously synthesized NGS^19^ exhibited a broad (002) peak at 23°, hinting extensive reduction of our starting material along with partial restoration of the graphitic structure. In contrast, the resultant eNGS evince high intensity, sharp (002) peak centred at 24° along with a small (100) peak at 43°, suggesting the formation of highly exfoliated and crystalline eNGS. Additionally, the interlayer spacing was also seen to increase slightly from 0.354 nm (NGS)^19^ to 0.365 nm for eNGS. These results demonstrate the successful synthesis of high quality, crystalline, and well exfoliated nanomaterial, which could be attributed to the irreversible reduction of eNGS *via*. electrochemical route.

Additionally, BET specific surface area of the electrochemically synthesized nitrogen doped graphene nanosheets (eNGS) was calculated from the nitrogen adsorption-desorption isotherms at 77 K to determine the porous nature of the nanomaterial (Figure S3). This nitrogen adsorption-desorption isotherm of eNGS exhibit the type IV adsorption isotherm phenomenon. The appearance of hysteresis in the isotherm depicts the irreversible desorption traits which are usually noted in nanomaterials which are mesoporous in nature. The SBET for eNGS was calculated to be 169 m^2^g^−1^ with a pore volume of 1.43 cm^3^g^−1^.

**Figure 3:**
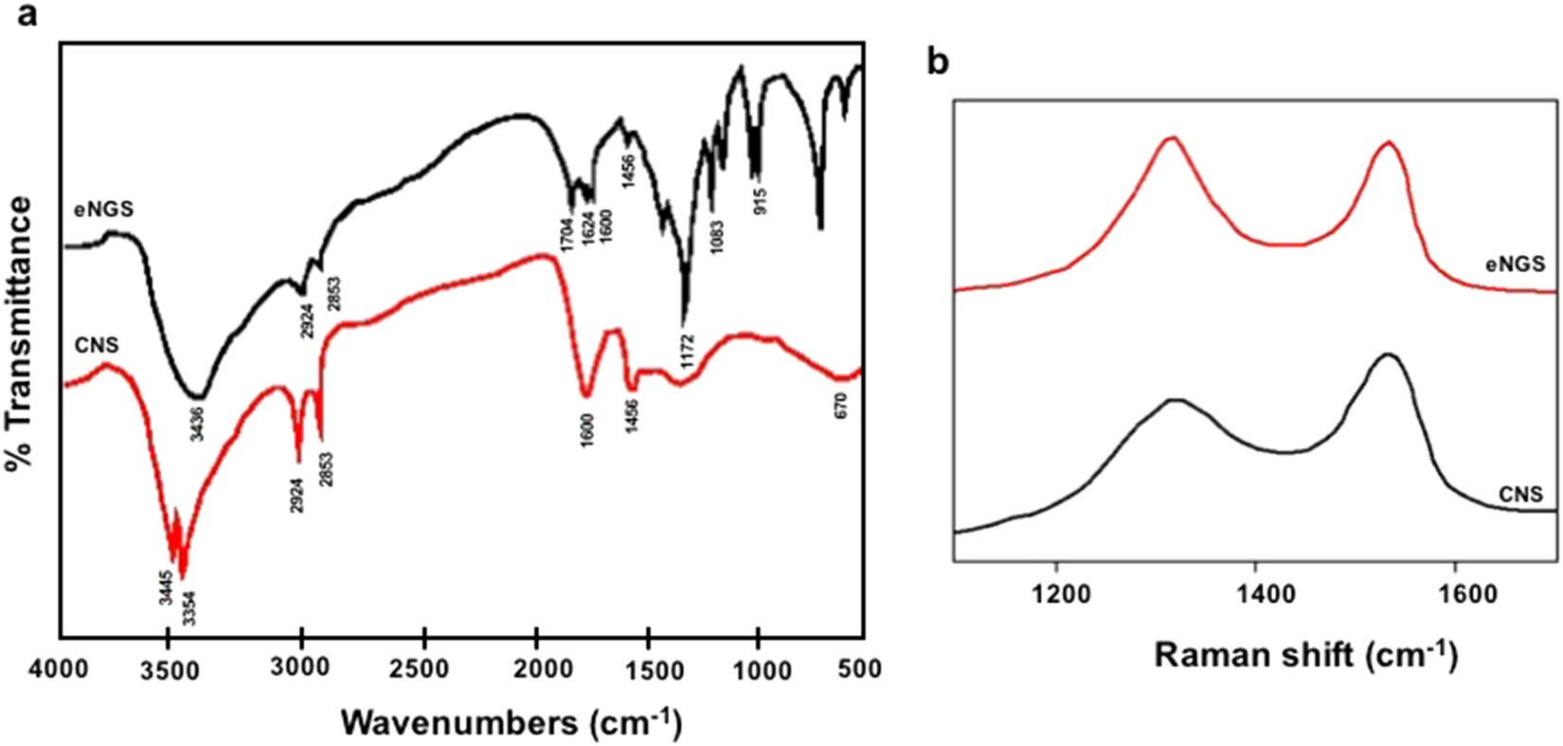
(**a**) FTIR of CNS and eNGS (**b**) Raman spectra (514.5 nm) of CNS and eNGS.

Raman and FTIR spectroscopy were used to confirm the reduction of eNGS. FTIR was employed to examine the extent of CNS reduction. Figure 3a displays the FTIR transmittance spectra of the exfoliated CNS and eNGS. The spectrum of CNS shows the presence of oxygen containing functional groups, C=O and C-O at peak positions 1703 cm^−1^ and 1172 cm^−1^, respectively. The presence of fingerprint shoulder peak at 1456 cm^−1^ which was attributed to triazine, was seen in both CNS and eNGS, suggesting that the structural framework of the material generating from melamine was retained. The disappearance of absorption bands for O=CO vibrations at 1624, 1227, and 1283 cm^−1^ further prove that the as- synthesized eNGS was devoid of oxygen containing moieties and thus was successfully reduced. Moreover, a sharp increase in peak intensities at 2920 cm^−1^ and 2851 cm^−1^ attributed to asymmetric CH_2_ stretching vibrations along with an enhanced peak at 1604 cm^−1^ (C=C stretching) confirm the reduction of oxygen functionalities and restoration of sp^2^ structure (π-conjugation), respectively^28^. Figure 3b depicts the Raman measurements of the as-synthesized CNS and eNGS. Raman spectrum of CNS displays a broadened G-band (~1590 cm^−1^) arising from sp^2^ C-atoms and D-band (~1365 cm^−1^) which is attributed to the presence of disorder and boundaries. The prominent D-band also represents a decrease in the size of in-plane sp^2^ domains due to the presence of large number of oxygen containing functional groups. In case of eNGS, both G-band and D-band were retained, however the intensities of D-band were higher than the D-band of CNS. the I_D_/I_G_ ratio of eNGS was seen to increase to 0.89 (from 0.83, I_D_/I_G_ ratio for CNS). This altercation in the intensity ratios of D- to G- bands is ascribed to the increased number of defects in eNGS relative to CNS, further confirming the successful deoxygenation of CNS to form eNGS^32, 33^. These alterations imply a decrease in the overall size of the sp^2^ domains due to the successfully reduced eNGS, which can be further supported by the formation of new graphitic domains as was also proven by the XRD data.

**Figure 4:**
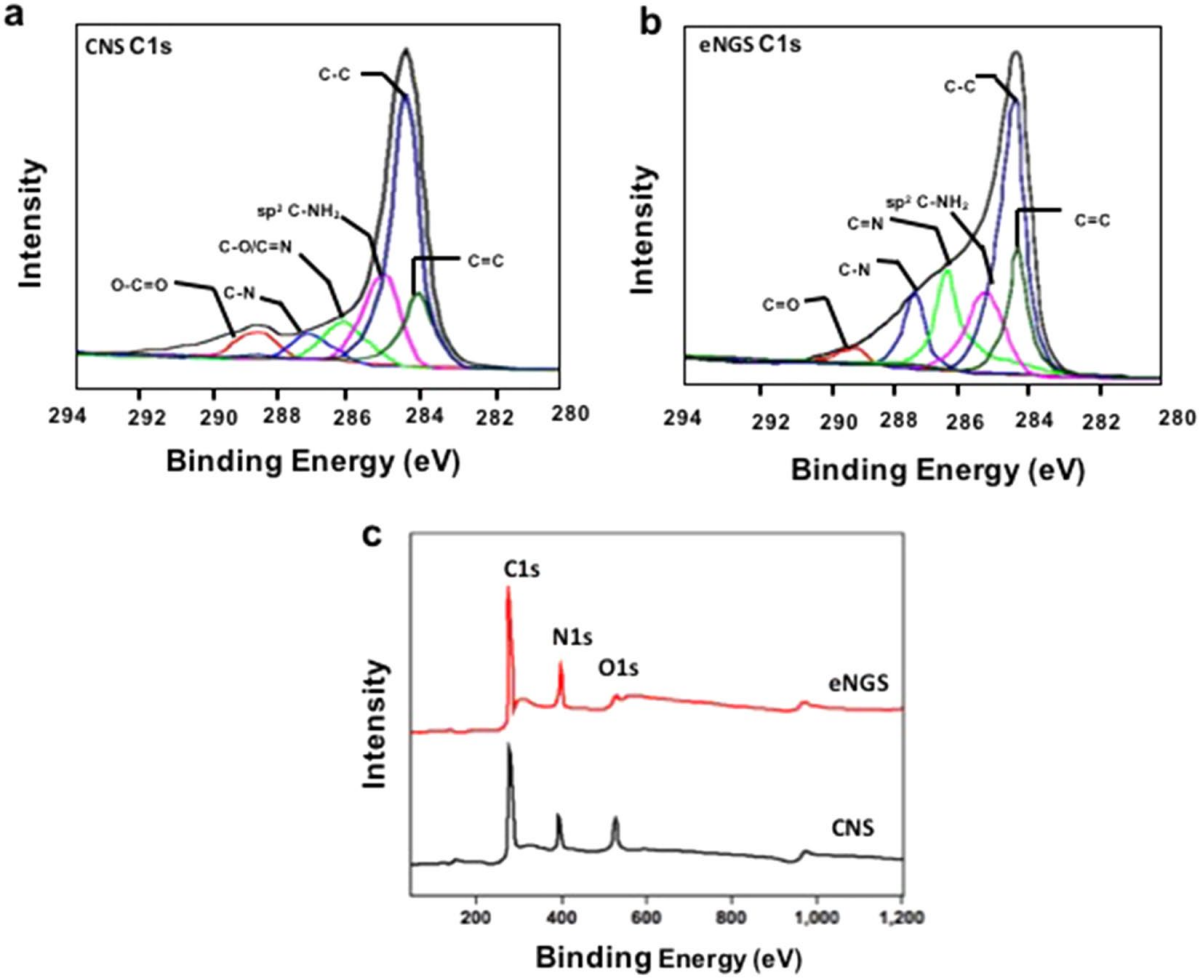
(**a**) C1s spectra of CNS, (**b**) C1s spectra of eNGS, and (**c**) Survey XPS spectra of CNS and eNGS.

Furthermore, XPS measurements were carried out to establish a more detailed version of the eNGS structure and to reveal the chemical bonding states pre- and post- electrochemical reduction. The C1s peak for the untreated CNS (Fig. 4a) were categorized into six distinctive peaks, corresponding to C-C (284.5 eV), C=C (284.1 eV), C-O/C=N (286.2 eV), sp^2^ C-NH_2_ (285.6 eV), O-C=O (288.8 eV), and C-N (287.0 eV)^19, 34–36^. In contrast to C1s peaks of CNS, its reduced counterpart, eNGS C1s displays a small decrease in the peak intensities for C-C and C=O, confirming decomposition of oxygen functionalities (Fig. 4b). Moreover, peaks assigned to C-O/C=N remained unaltered, thereby suggesting the incorporation of triazine ring (originating from melamine) in the eNGS framework^19^. Additionally, a marginal decrease in the peak intensities of sp^2^ C-NH_2_ were observed, which could be ascribed to the electrochemical treatment of the material, resulting in the intercalation of the melamine groups in the graphene layers. The XPS results are in agreement with FTIR data, confirming the presence of sp^2^ carbon structure and triazine structures in both CNS and eNGS.

**Figure 5:**
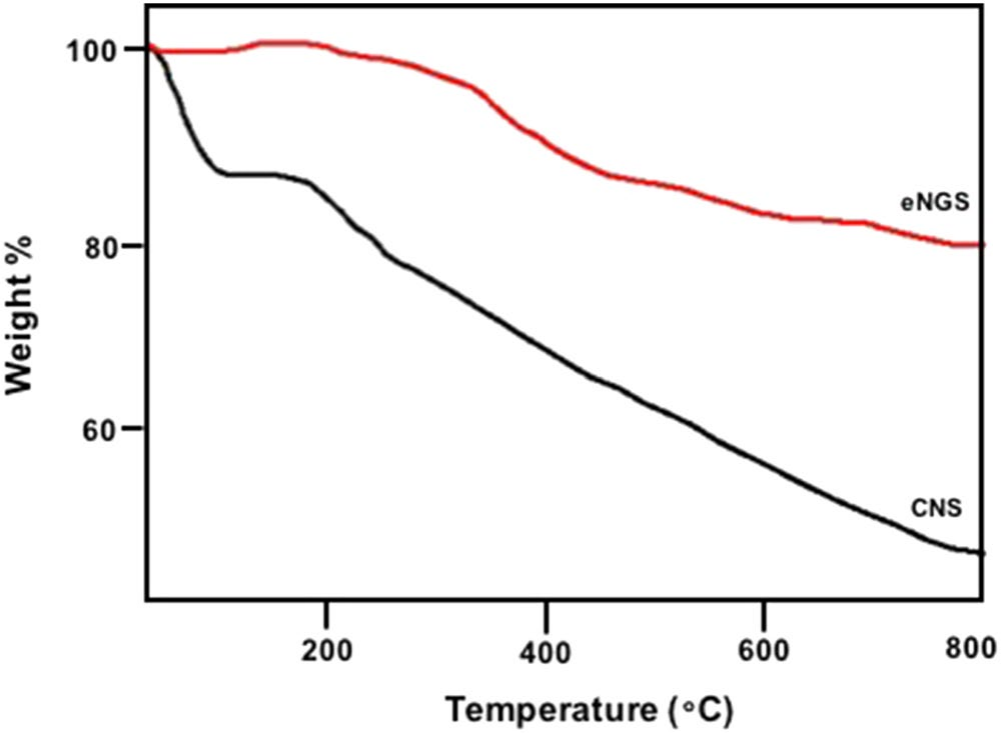
TGA of CNS and eNGS under N_2_ atmosphere.

We, additionally investigated the thermal stability of the as-synthesized eNGS using TGA, in N_2_ atmosphere (Fig. 5). In CNS, most of the weight loss occurred between 50 °C to 600 °C, which could be due to the release of adsorbed water molecules and labile functional groups.

However, eNGS exhibits enhanced thermal stability where only a 16% weight loss was observed below 800 °C. This major mass loss can be attributed to the removal of oxygen containing functional groups due to electrochemical treatment^37^. These results were further validated by the CHNO elemental analysis (Supplementary Figure S4).

These results signified that the as-synthesized eNGS are nitrogen rich graphene nanosheets which comprise of large number of edge plane defects, large surface-volume ratio, and very few residual oxygen functionalities, which would plausibly render the material highly suited for exceptional electrocatalytic performance^38, 39^. We therefore, were keen on examining the amperometric responses of our material for sensing DA.

**Figure 6:**
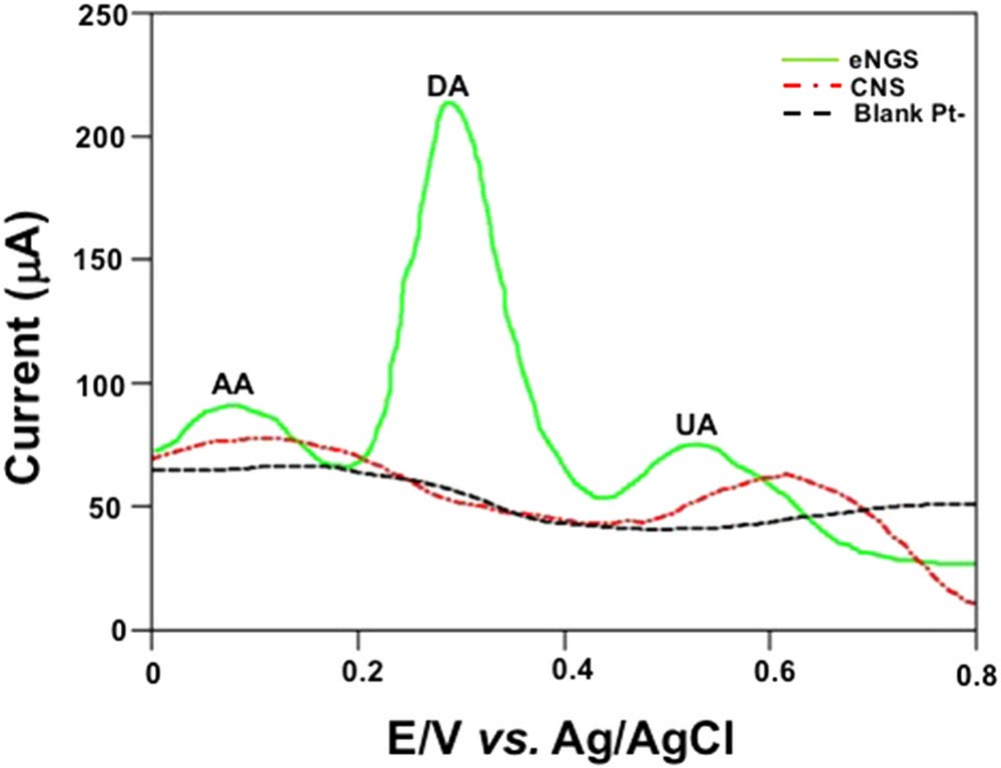
Bare Pt-, Pt-/CNS, and Pt-/eNGS in 100 µM DA (in 0.1 M PBS/pH 7.4) in the presence of interferences (150 µM AA and UA) at 50 mV/s.

In order to evaluate the sensing potential of an electrocatalyst, they are usually assessed by measuring their current responses after the subsequent addition of the analyte of interest and the interfering agents to be examined. eNGS were applied for determination of DA from human urine samples. The urine sample was diluted 2x with 0.1 M PBS (pH 7.4) prior to measurements. Linear sweep voltammogram (LSV) responses of bare Pt-, CNS, and eNGS modified electrodes (Fig. 6). The behaviour of Pt-/eNGS modified electrode was examined by recording the LSV for DA (100 µM DA in 0.1 M PBS) and was compared with Pt-/CNS modified electrode. An enhanced, widely separated and distinct current response at relatively lower potentials for DA in the presence of interferences, namely AA and UA, (DA – 0.25 V, AA – 0.11 V, and UA – 0.52 V) with respect to a broad, diffused hump for DA in the presence of AA in case of Pt-/CNS clearly testify the ameliorated electrocatalytic response of eNGS over CNS. The improved electrocatalytic response of eNGS towards DA sensing in the presence of UA and AA could be due to idiosyncratic electronic attributes of our nanoelectrocatalyst, leading to an increase in electron transfer rate due to improved conductivity. This trend of enhanced sensitivity and selectivity could possibly be attributed to the presence of larger amounts of edge plane defects^36, 40^, surface-volume ratio^41, 42^, and N-enrichment^42–45^ along with a notable improvement in electrical conductivity (Supplementary Figure S5), plausibly credited to the restoration of the graphitic structure. Furthermore, long term DA oxidation on Pt-/CNS and Pt-/eNGS was examined by chronoamperometry at a near peak-to-peak applied potential of 0.30 V. It was noted that CNS exhibited significant deactivation, which may be ascribed to electrode fouling. This poisoning of the electrode could be due to the presence of oxygenated/hydrogenated species^12, 45^. However, Pt-/eNGS chronoamperometry corroborated comparatively higher stability and current density for a longer time duration (Supplementary Figure S6). These observations further confirmed that eNGS possessed improved stability and electrocatalytic properties.

### Biocompatibility of eNGS

The as-synthesized eNGS was further evaluated for biological safety and toxicity if any. Therefore, now with a successfully synthesized eNGS boasting of enhanced sensitivity and selectivity in hand, we decided to go ahead and perform a suite of biocompatibility assays to determine the suitability of our material for future biological use.

In majority of the cases, blood-circulatory system is the most probable first complex to which an organ will be exposed. Therefore, a detailed understanding of the fundamental interaction pathway between blood, serum proteins, and the as-synthesized foreign nanoparticles would aid in an improved understanding of the potential perils that nanomaterials may pose and subsequently help in providing a direction for the safe design of nanomaterials. To begin with, we performed a dose-dependent blood cell aggregation analysis (Supplementary Figure S9). We examined the alterations in the morphology of RBCs post incubation with eNGS (Supplementary Figure S9). When compared with the biconcave, healthy RBCs in PBS (pH 7.4), the eNGS treated RBCs exhibited aberrant morphological alterations at lower concentrations (10 μg/ml and 50 μg/ml). However, at higher concentrations of eNGS (100 μg/ml), insignificant hemagglutination was observed. These results signify that our nanoelectrocatalyst is hemocompatible.

### Effect of eNGS on FOB

Living systems have its self defence mechanism against any foreign particle, drug or chemical. Body gives instant counter action against any ingested chemical and shows several behavioural changes as per the severity or toxicity. Thus, the functional observational battery (FOB) helps to find the instant functional and behavioural changes if any. In case of observations of home cage, eNGS (300 mg/Kg/*p.o*, once) did not show any behavioural abnormalities including convulsions, tremors, fasiculations, vocalizations, diarrhoea/defecation, change in posture, excessive urination, and respiration (Supplementary Figure S7). Moreover, on exterior site of home cage and handling, eNGS (300 mg/Kg/*p.o*, once) did not give any major behavioural alteration including lacrimation, salivation, body tone, piloerection, fur appearance, ptosis, and exophthalmia. For open-field activity, eNGS was also seen to have a neutral effect against surface righting, aerial righting, tail pinch and pinna reflexes, visual placing, palpebral closure, auditory response and hind limb foot splay. Thus, the upshot of FOB study represents no significant alteration on behavioural activity by the administration of eNGS as compared to control group (Supplementary Figure S8).

### Effect of eNGS on reduced glutathione

**Figure 7:**
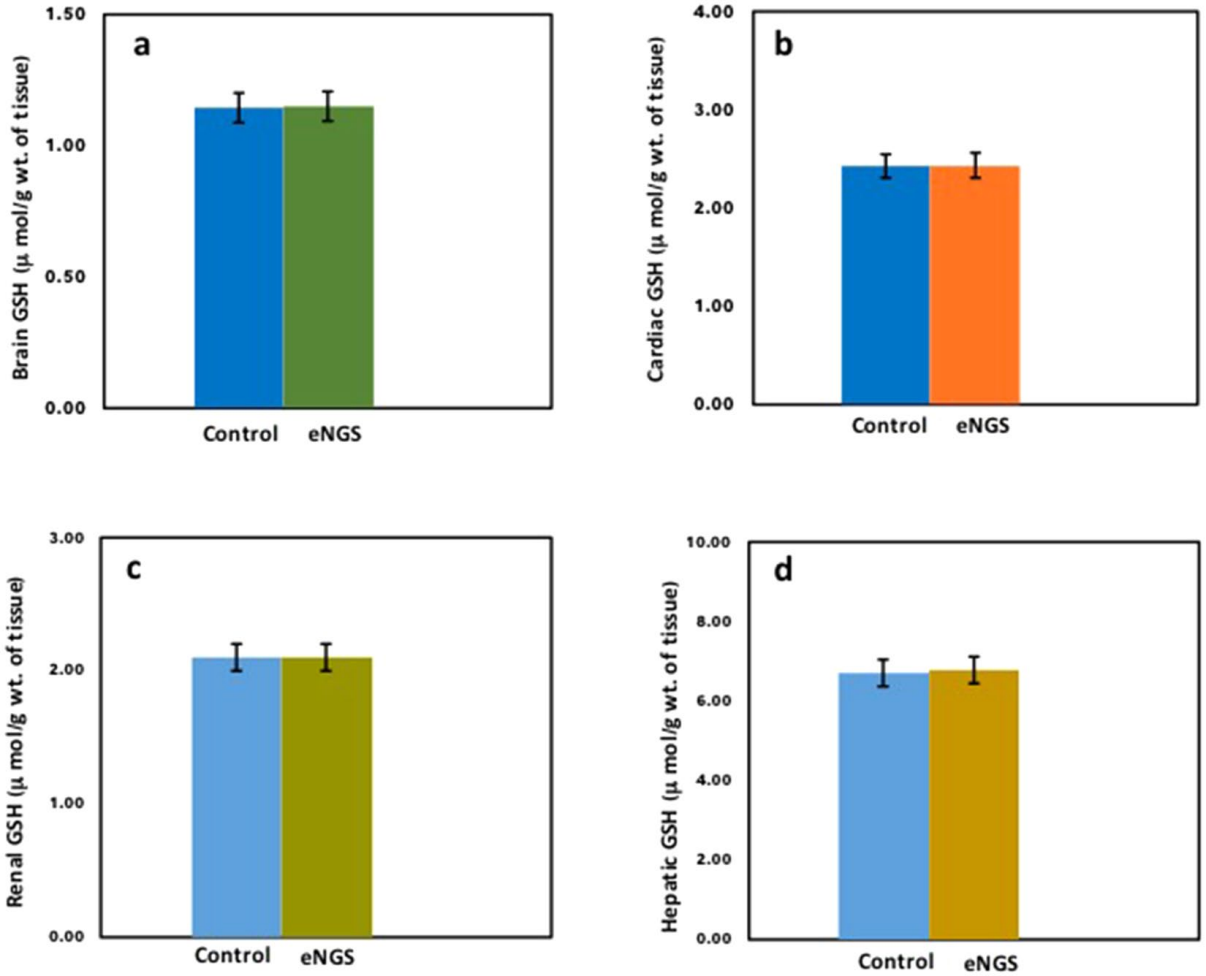
Effect of eNGS (300 mg/Kg/*p.o*, once) on glutathione concentration (μ mol/g wt. of tissue) against normal control rats. (**a**) glutathione concentration of brain, (**b**) glutathione concentration of heart, (**c**) glutathione concentration of kidney, (**d**) glutathione concentration of liver. Each group contain n = 3 rats and all values were represented as mean ± SD. Value of ‘*p’* less than 0.05 has been considered as statistically significant.

Oxidative stress is the major pitfall for several serious pathological conditions and altered signalling pathways. Indeed, animal bodies have the ability to counterbalance these inevitable cellular by-products by self-inflicted natural antioxidants including glutathione (GSH). Glutathione is the master antioxidant and helps to keep redox-sensitive active sites of enzyme in the necessary reduced states. GSH is the regular defence system of body which directly condenses reactive free radicals, boosts immune function, and diminishes the vulnerability of tissue damage by alleviating oxidative stress^42^. eNGS (300 mg/Kg/*p.o*, once) administration resulted in no significant alteration in reduced glutathione levels, as compared to normal control. Inconsequential (*p* < *0.05*) variation in tissues including heart, brain, liver, and kidney was observed in rats administered with eNGS with respect to normal control (Fig. 7).

### Effect of eNGS on histology

**Figure 8:**
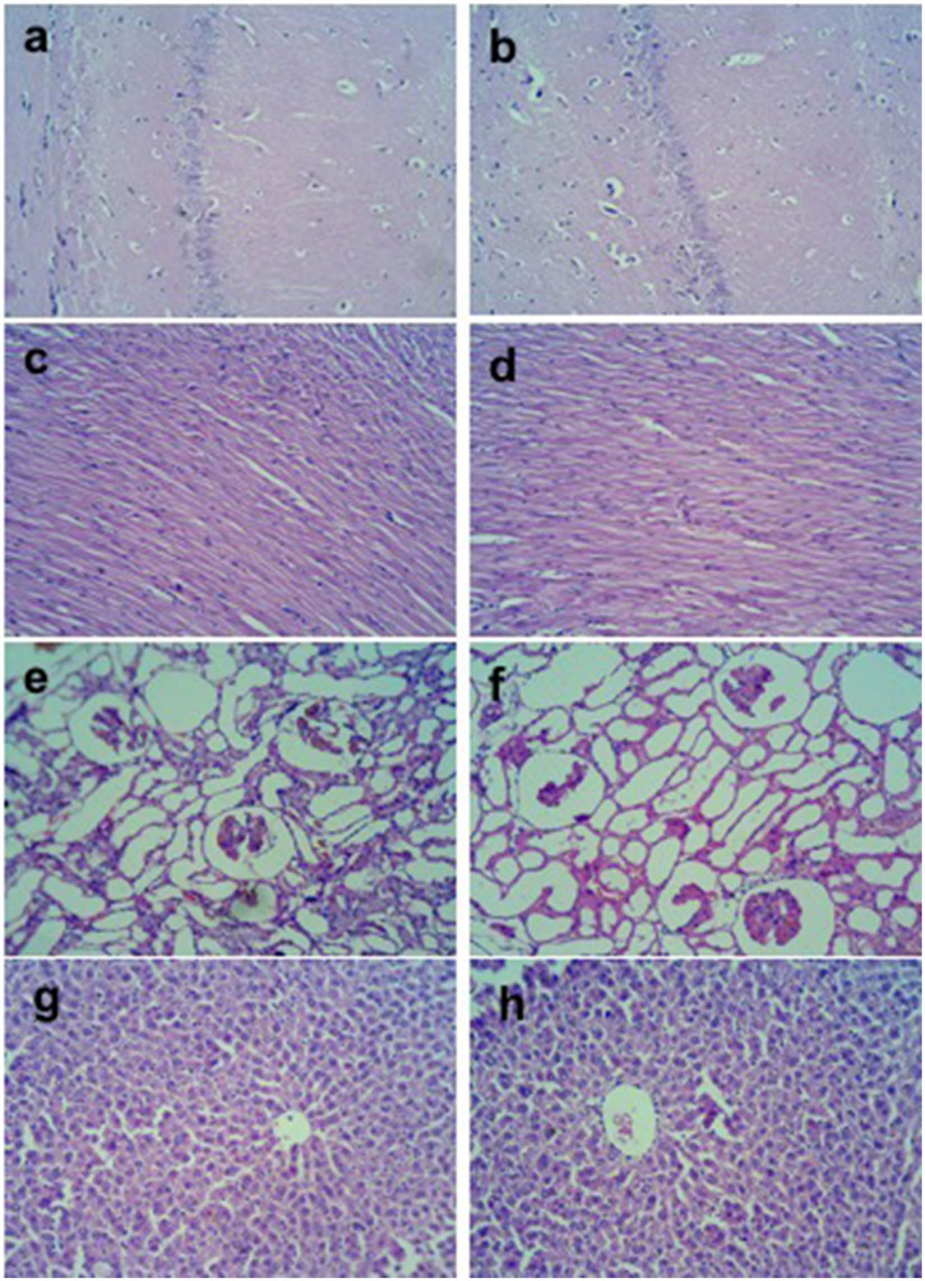
Histological sections of brain (a-Control, b-eNGS), (c-Control, d-eNGS) heart, (e-Control, f-eNGS) kidney, and (g-Control, h-eNGS) liver from each group. Photomicrographs of each tissue of normal control and eNGS rats were taken at 400x.

Photomicrographs of different tissues from both the groups of rats (Normal control and eNGS) were examined at 400x. Histological evaluations of all the organs revealed insignificant structural changes in case of eNGS, as compared to normal control group for all organs. This observation is based on the unsubstantial structural variations observed in myocytes nuclei and myofibrillar for heart, negligible structural changes and cell death in photomicrographs of hippocampus (CA1) section for brain, no disruption in central vein and hepatocytes for liver and similarly no major alteration in parenchyma, tubules and glomeruli for kidney when compared with the histological section of same organs of normal control rats (Fig. 8).

We, thus concluded that the behavioural patterns observed after 30 min of eNGS administration clearly revealed the negligible alteration when exposed to the prepared material. Therefore, on the basis of favourable results achieved for eNGS, we decided to also analyse the biochemical and structural changes it would confer to the vital organs. Interestingly, it was noted that eNGS contact to rat body’s organs didn’t show any alterations in GSH level which displays its non-toxic and non-interfering presence in body defence system. Similar consequences of GSH level in all tissues as compared to normal tissue clearly indicate the non-toxic nature of eNGS in terms of oxidative stress in vital organs. Furthermore, structural changes have been evaluated by studying the histology of vital organs but no significant changes were seen in any photomicrographs as compared to normal tissues. These findings thereby establish eNGS as an exceptional biocompatible material with promising potentials in complex biological systems.

Furthermore, we performed a detailed biochemical analysis of eNGS. Since, pro-inflammatory cytokines (interleukin-6, IL-6; interleukin 10, IL-10; tumour necrosis factor alpha, TNFα), lipid peroxidation (thiobarbituric acid reactive substances, TBARS) and cholesterol levels are the most vulnerable biochemical parameters and can result in serious pathological predispositions, we chose to examine all these above mentioned factors as an extension of the biocompatibility parameters for our eNGS. These biochemical parameters were analysed *via*. Sigma Plot® Version 11 by employing one-way ANOVA followed by Tukey’s test. Insignificant differences were observed for any biochemical parameter in case of eNGS treated group of animals as compared to normal control (Supplementary Table S10).

Moreover, cell cytotoxicity effects of eNGS were also studied on mouse fibroblast cells. The micrographs depicted effusive mushrooming of fibroblasts when incubated with 100 µg/ml of eNGS. However, CNS lead to loss in cell viability and disruption of the original, healthy spindle shaped fibroblast cells (Supplementary Figure S11). We thus inferred that our as-synthesized nanomaterial had very low cytotoxicity and unprecedented levels of biocompatibility, consequently presenting us with an array of promising potential applications in the field of medical biology. These findings thereby establish eNGS as an exceptionally biocompatible material with promising potentials in complex biological systems.

## Conclusion

Herein, we report a first time, one-step, eco-friendly, and rapid electrochemical synthesis of eNGS by reducing CNS. No reducing agents were employed, thereby banishing the potential contaminants which occur in case of chemical reduction. The as-synthesized ~1.2 nm thick eNGS, credited with unique properties, such as high surface-to-volume ratio, lightness, low charge transfer resistance, and improved electrochemical properties, was employed for sensitive and selective real-time detection of DA from human urine sample. We further successfully carried out *in vitro* studies on Wistar albino rats of either sex for the determination of the biocompatibility of eNGS. It was evidently corroborated that eNGS was an exceptionally biocompatible nanomaterial with no detrimental effects on the GSH levels in tissues of vital organs. Pictomicrographs of different tissues from both the groups of rats (normal control and eNGS), also revealed no substantial difference for myocytes nuclei and myofibrillar for heart, hippocampus (CA 1) section of brain, central vein and hepatocytes for liver and similarly no major alterations in parenchyma, tubules, and glomeruli for kidney were seen. This study therefore not only establishes a facile, rapid, and eco-friendly route for the synthesis of highly sensitive, selective, and stable nanoelctrocatalysts, but also proffers a novel nanomaterial with remarkably high biocompatibility for the development of mighty platforms with promising potentials in the field of modern biosensing and other complex biological systems. However, nitrogen doping without the involvement of any toxic reagents (due to electrochemical reduction procedures) play a key role in rendering our nanomaterial highly biocompatible in nature, the plausible physicochemical mechanisms, and/or determinants behind eNGS displaying such exceptional biocompatibility is still under investigation.

## Electronic supplementary material

Electrochemically synthesized highly crystalline nitrogen doped graphene nanosheets with exceptional biocompatibility
