# Supplementary material for: Electrochemically synthesized highly crystalline nitrogen doped graphene nanosheets with exceptional biocompatibility
Source: Sci Rep. 2017 Apr 3;7:537. doi: 10.1038/s41598-017-00616-8 (PMC5428811; doi:10.1038/s41598-017-00616-8)
Supplement: Supplementary file 1 — Electrochemically synthesized highly crystalline nitrogen doped graphene nanosheets with exceptional biocompatibility [file 41598_2017_616_MOESM1_ESM.doc]

**Supplementary Information**

**Electrochemically synthesized highly crystalline nitrogen doped graphene nanosheets with exceptional biocompatibility**

*Deepa Suhag^a^, Arun Kumar Sharma^b^, Satyendra K. Rajput^b^, Gajender Saini^c^, Sandip Chakrabarti^d^ and Monalisa Mukherjee^a^**

*^a^* Biomimetic and Nanostructured Materials Research laboratory, Amity Institute of Biotechnology, Amity University Uttar Pradesh, Sector-125, Noida-201303, India.

*^b^* Amity Institute of Pharmacy, Amity University Uttar Pradesh, Sector-125, Noida-201303, India.

*^c^* Advance Instrumentation Research Facility, Jawaharlal Nehru University, New Delhi, India.

*^d^* Amity Institute of Nanotechnology, Amity University Uttar Pradesh, Sector-125, Noida-

201303, India.

**S1**

**Materials**

Glycerol, melamine, dopamine and uric acid were procured from Sigma-Aldrich. Concentrated sulphuric acid (98%) was purchased from Merck, Germany. All chemicals except melamine were used as received without further purifications. 5-10 ml of mid-stream urine was collected from normal individual to study dopamine sensing. The study was approved by the institution’s ethical committee and informed consent was obtained from the human volunteers who readily agreed to give their urine sample. The experiments were performed in compliance with the relevant laws and institutional guidelines.

**Chemical composition**

Fourier transform infrared spectroscopy (FTIR) spectra were recorded using KBr pellets on a Nicolet-5DX FTIR Spectrophotometer. The spectra were measured from 500 to 4000 cm^-1^. Raman spectroscopy analyses were performed using a confocal micro-Raman LabRam HR instrument (Horiba Scientific) in backscattering geometry with a CCD detector at 514.5 nm Ar laser and a 100 x objective mounted on an Olympus optical microscope. Calibration was initially done using an internal silicon reference at 520 cm^-1^ which gave a peak position resolution of less than 1 cm^-1^. X-ray Photoelectron Spectroscopy (XPS) measurements were performed on a Kratos Axis Ultra Photoelectron Spectrometer which uses Al Kα (1253.6 eV) X-rays. Curve fitting and background subtraction were performed using Casa XPS version 2.2.73 software. CHN and O Elemental analysis were performed by Perkin-Elmer 2400 Series CHNS/O Analyser. Thermogravimetric analysis (TGA) studies were performed using Mettler-Toledo, TG850 in N_2_ atmosphere (flow rate = 50 ml/min) in the temperature range 25 – 800 °C (heating rate = 5 °C/min).

**Morphology**

Transmission Electron Microscopy (TEM) and High Resolution Transmission Electron Microscopy (HRTEM) were taken on a JEOL, JEM-2100F electron microscope at an acceleration voltage of 200 kV. Samples were prepared by drop casting the sample dispersion material onto a carbon coated copper grid followed by drying at room temperature. Atomic force microscopy (AFM) images were obtained by Dimension 3000 with tapping mode to obtain the morphology of the samples. AFM samples were prepared by coating eNGS on freshly cleaved mica sheets.

**Statistical Analysis**

For FOB calculation, values were expressed in comparative median of each group, statistical analysis was performed by using Kruskal-Wallis Way Analysis of Variance on Ranks. Effect of nanomaterial administration, as compared with control group and hindlimb foot splay activity were recorded. The data were expressed as mean ± standard deviation (SD) and analyzed by using one-way analysis of variance (ANOVA) followed by Tukey’s test. The *p* value of < 0.05 is considered as statistically significant.

**Cell Culture**

McCoy mouse fibroblast cells were utilized for analysing the cytotoxicity of our as-synthesized eNGS. The cells were cultured and maintained in 25 cm^2^ tissue culture flasks in DMEM media (Sigma) containing 10 % of FBS (Sigma), 10 µg/ml ciprofloxacin, and 5 % CO_2_.

Synthesis of CNS:

In a typical synthesis,^1^ 0.5 g of purified melamine was added to 10 ml of glycerol and stirred until melamine was dissolved in glycerol followed by the addition of 10 ml of 98% sulphuric acid under vigorous stirring. The mixture was transferred to a 40 ml PTFE lined stainless steel autoclave and heated at 180 ^o^C for 4h under autogeneous pressure. The resulting product (CNS) turned into black colored powder which was washed with ethanol followed by deionized (DI) water. The synthesized CNS was dried at 50 ^o^C in hot air oven and was further treated hydrothermally.

S2

**AFM**

At present, Atomic force microscopy (AFM) is the foremost tool which allows the definitive recognition of single layer crystals.^2^ Figure S2 displays the AFM of the exfoliated eNGS. To begin with, Pt- electrode coated with the as-synthesized eNGS was immersed repeatedly in DI water. This was done to get rid of CNS or any remnant salts from the electrode surface. This was followed by gentle stirring of the eNGS coated electrode in DI water to disperse the loosely bound eNGS. A piece of mica was then immersed into the DI water containing dispersed eNGS which was further utilized for AFM characterizations. As is seen in figure S2, the electrochemically exfoliated eNGS are flat, ~1.2 nm thick sheets with a lateral dimension of ~ 120 nm.

**Tapping mode AFM images of (a) uncoated mica sheets, and (b) the exfoliated eNGS with height profiles**

**S3**

**BET isotherm of the eNGS at 77 K**

**S4**

**Carbon, Hydrogen, Nitrogen, and Oxygen percentages from CNS and NGS**

| Sample Name | C atom % | O atom % | N atom % | H atom % | C/O ratio | C/N ratio |
| --- | --- | --- | --- | --- | --- | --- |
| CNS | 81.69 | 13.12 | 1.68 | 3.51 | 6.2 | 48.6 |
| eNGS | 88.30 | 7.72 | 2.2 | 1.78 | 11.44 | 40.14 |

**S5**

**I-V curves for CNS, NGS, and eNGS**

**
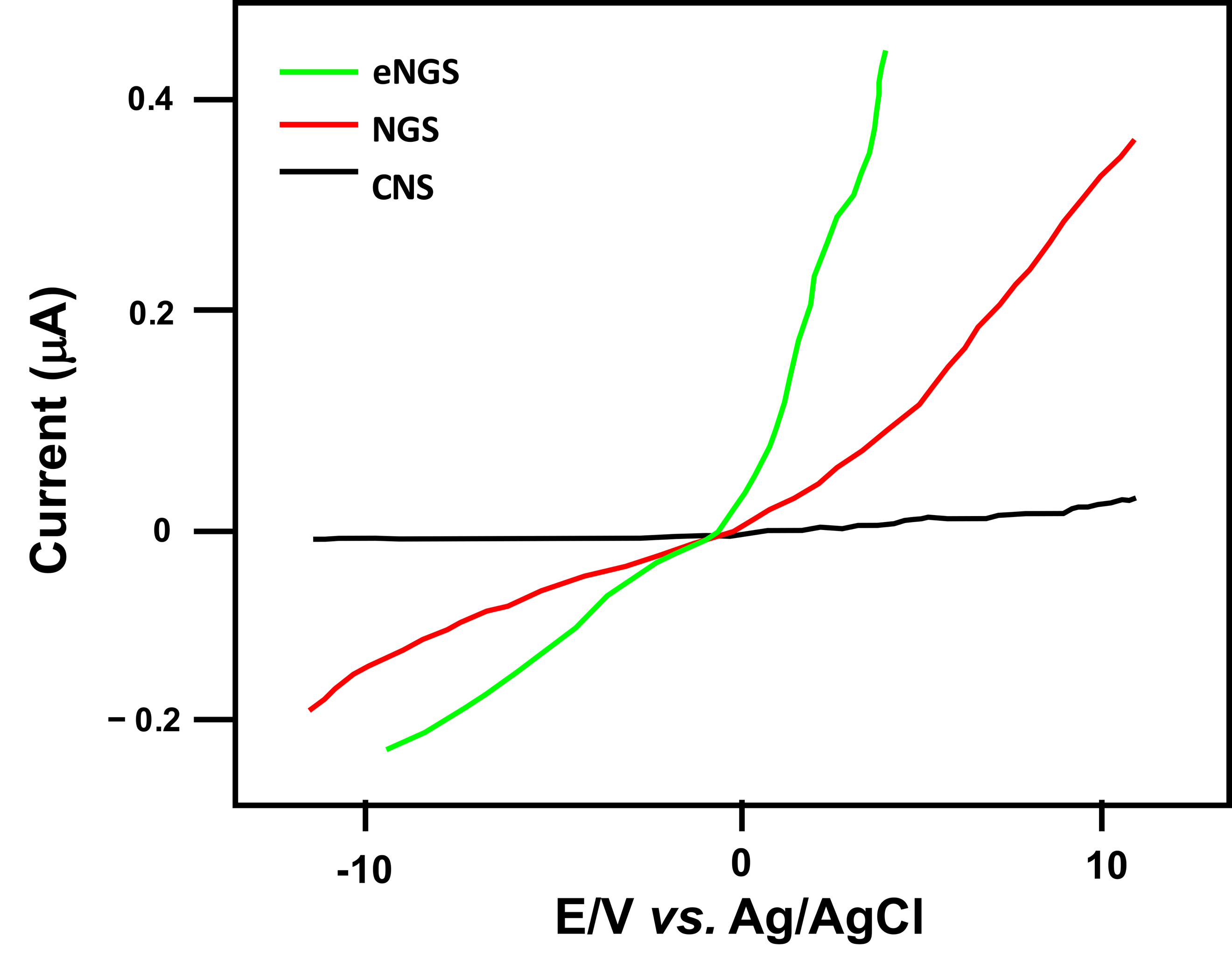
**

**S6**

**Chronoamperometric curves of Pt-/eNGS vs Pt-/CNS. Applied potential: 0.30 V**

**S7**

**Observations of home cage for eNGS (300 mg/Kg/*p.o*, once) vs. Normal control**

**S8**

Table 1

| Categories | Normal Control | C-1 | | | | N-1 | | | N-2 | | |
| --- | --- | --- | --- | --- | --- | --- | --- | --- | --- | --- | --- |
|  |  | Rat-1 | | Rat-2 | Rat-3 | Rat-1 | Rat-2 | Rat-3 | Rat-1 | Rat-2 | Rat-3 |
| Home Cage | | | | | | | | | | | |
| Spontaneous activity level | 3 | 3 | 3 | | 3 | 3 | 3 | 3 | 3 | 3 | 3 |
| Posture | 2 | 2 | 2 | | 2 | 2 | 2 | 2 | 2 | 2 | 2 |
| Respiration | 3 | 3 | 3 | | 3 | 3 | 3 | 3 | 3 | 3 | 3 |
| Convulsions | A | A | A | | A | A | A | A | A | A | A |
| Tremors | A | A | A | | A | A | A | A | A | A | A |
| Fasiculations | A | A | A | | A | A | A | A | A | A | A |
| Tonus | A | A | A | | A | A | A | A | A | A | A |
| Clonus | A | A | A | | A | A | A | A | A | A | A |
| Vocalization | A | A | A | | A | A | A | A | A | A | A |
| Straubs tail | A | A | A | | A | A | A | A | A | A | A |
| Writhing | A | A | A | | A | A | A | A | A | A | A |
| Retropulsion | A | A | A | | A | A | A | A | A | A | A |
| Diarrhoea | A | A | A | | A | A | A | A | A | A | A |
| Hand held | | | | | | | | | | | |
| Excitation | 2 | 2 | | 2 | 2 | 2 | 2 | 2 | 2 | 2 | 2 |
| Salivation | 0 | 0 | | 0 | 0 | 0 | 0 | 0 | 0 | 0 | 0 |
| Lacrimation | 0 | 0 | | 0 | 0 | 0 | 0 | 0 | 0 | 0 | 0 |
| Piloerection | A | A | | A | A | A | A | A | A | A | A |
| Fur appearance | A | A | | A | A | A | A | A | A | A | A |
| Ptosis | A | A | | A | A | A | A | A | A | A | A |
| Exophthalmia | A | A | | A | A | A | A | A | A | A | A |
| Open Cage | | | | | | | | | | | |
| Supported rears | 5 | 5 | | 5 | 5 | 5 | 5 | 5 | 5 | 5 | 5 |
| Unsupported rears | 0 | 0 | | 0 | 0 | 0 | 0 | 0 | 0 | 0 | 0 |
| Spontaneous activity level | 3 | 3 | | 3 | 3 | 3 | 3 | 3 | 3 | 3 | 3 |
| Gait | 1 | 1 | | 1 | 1 | 1 | 1 | 1 | 1 | 1 | 1 |
| Posture | 2 | 2 | | 2 | 2 | 2 | 2 | 2 | 2 | 2 | 2 |
| Arousal | 4 | 4 | | 4 | 4 | 4 | 4 | 4 | 4 | 4 | 4 |
| Convulsions | A | A | | A | A | A | A | A | A | A | A |
| Straubs tail | A | A | | A | A | A | A | A | A | A | A |
| Writhing | A | A | | A | A | A | A | A | A | A | A |
| Retropulsion | A | A | | A | A | A | A | A | A | A | A |
| Stereotypy | A | A | | A | A | A | A | A | A | A | A |
| Diarrhoea | A | A | | A | A | A | A | A | A | A | A |
| Auditory response | 3 | 3 | | 3 | 3 | 3 | 3 | 3 | 3 | 3 | 3 |
| Somatosensory respose | 3 | 3 | | 3 | 3 | 3 | 3 | 3 | 3 | 3 | 3 |
| Visual approach | P | P | | P | P | P | P | P | P | P | P |
| Olfactory response | P | P | | P | P | P | P | P | P | P | P |
| Pinna reflex | P | P | | P | P | P | P | P | P | P | P |
| Extensor reflex | P | P | | P | P | P | P | P | P | P | P |
| Palpebral reflex | P | P | | P | P | P | P | P | P | P | P |
| Visual placing | P | P | | P | P | P | P | P | P | P | P |
| Surface righting | P | P | | P | P | P | P | P | P | P | P |
| Aerial righting | P | P | | P | P | P | P | P | P | P | P |
| Pupil reaction | P | P | | P | P | P | P | P | P | P | P |
| Tail pinch response | P | P | | P | P | P | P | P | P | P | P |
| Urination spots | P | P | | P | P | P | P | P | P | P | P |
| Muscle tone | P | P | | P | P | P | P | P | P | P | P |

**S9**

**Optical micrographs of RBCs in the presence of (a) Normal Saline (control), (b) eNGS-10 μg/ml, (c) eNGS-50 μg/ml, and (d) eNGS-100 μg/ml**

**
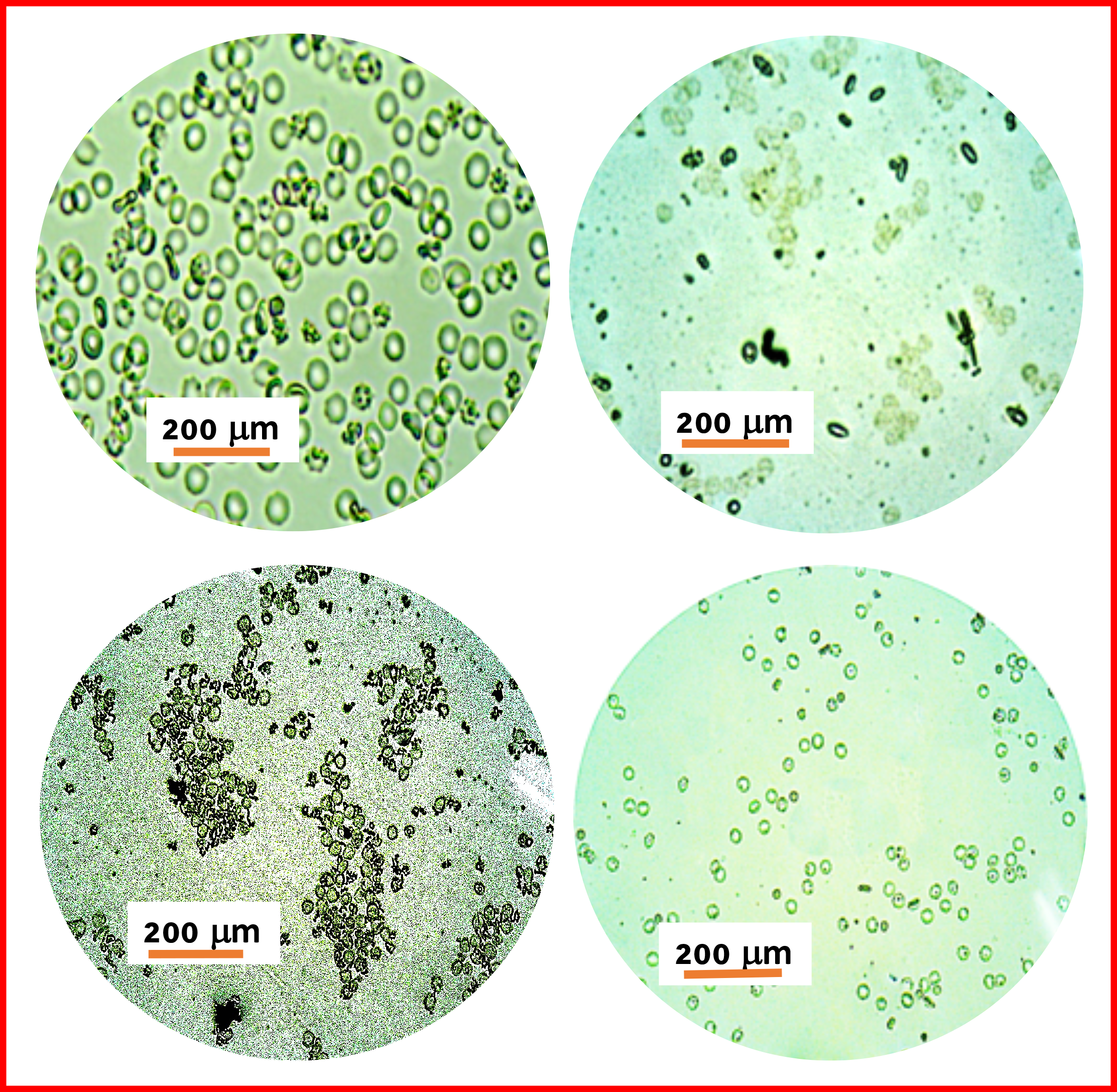
**

**Hemolysis Assay**

Hemolysis assay was performed to examine the *in vitro* hemocompatibility of our as-synthesized eNGS in order to explore their application potentials in the field of biomedical devices including injectable graphene-related particles. Fresh anticoagulated blood was collected from healthy mouse and diluted with 10 ml of normal saline (0.9 % concentration) at v/v ratio of 4:5. eNGS was then washed twice with normal saline. 0.1 ml diluted blood was successively added to eNGS-saline suspension.

**S10**

**Table 2: Findings of various biochemical parameters evaluation**

| **Sr. No** | **Biochemical Parameters**  **(Serum)** | **Normal Control Group (mean ± SD)** | **eNGS Treated Group (mean ± SD)** |
| --- | --- | --- | --- |
| **1** | Interleukin 6 (IL-6); pg/ml | 27.10 **±** 0.23 | 26.52 **±** 1.23 |
| 2 | Interleukin 10 (IL-10); pg/ml | 18.01 **±** 0.22 | 17.93 **±** 0.43 |
| 3 | Tumour necrosis factor alpha (TNFα); pg/ml | 13.27 **±** 0.29 | 13.79 **±** 0.29 |
| 4 | Thiobarbituric acid reactive substances (TBARS); µ mol/g wt. of tissue | 56.56 **±** 0.89 | 56.81 **±** 0.04 |
| **Lipid profile** | | | |
| 5 | Total cholesterol (TC); mg/dl | 120.93**±** 1.54 | 121.50**±**1.91 |
| 6 | High-density lipoproteins (HDL); mg/dl | 15.50 **±** 0.79 | 14.99 **±** 0.68 |
| 7 | Low-density lipoproteins (LDL); mg/dl | 15.28 **±** 0.19 | 15.43 **±** 0.80 |
| 8 | Triglyceride (TG); mg/dl | 127.86**±** 1.05 | 127.43**±**1.34 |

**S11**

**Optical micrographs of McCoy mouse fibroblast cells after 96 h (a) Normal Control, (b) CNS and (c) eNGS**

References:

1. Wang et al., J. Mater. Chem. A, 2014, 2, 2390.
2. Novoselov et al., Proc. Natl. Acad. Sci. U.S.A. 2005, 102, 10451.
